# The Distribution Pattern of the Neurovascular Structures for Anterior Ankle Arthroscopy to Minimize Structural Injury: Anatomical Study

**DOI:** 10.1155/2018/3421985

**Published:** 2018-05-15

**Authors:** Anna Jeon, Chang Min Seo, Je-Hun Lee, Seung-Ho Han

**Affiliations:** ^1^Department of Anatomy, College of Medicine, Chung-Ang University, Seoul, Republic of Korea; ^2^Anatomy Laboratory, College of Sports Science, Korea National Sport University, Seoul, Republic of Korea

## Abstract

**Introduction:**

The aim of this study was to investigate entry points for anterior ankle arthroscopy that would minimize the risk of neurovascular injury.

**Methods:**

Thirty-eight specimens from 21 Korean cadavers (age range from 43 to 92 years, mean age of 62.3 years) were used for the study. For the measurements, the most prominent points of the lateral malleolus (LM) and the medial malleolus (MM) were identified before dissection. A line connecting the LM and MM, known as the intermalleolar line, was used as a reference line. We measured 14 variables passed on the reference line.

**Results:**

This study found that the nerves were located at 40.0%, 50.0%, and 82.0% of the reference line from the lateral malleolus. We also found that the arteries were located at 22.0%, 35.0%, and 60% of the reference line from the lateral malleolus.

**Discussion:**

If all the variables are combined (nerves, arteries, and veins), then there is no safety zone for anterior portal placement. Therefore, we recommend that surgeons concentrate primarily on the arteries and nerves in the clinical setting.

## 1. Introduction

Arthroscopy can accurately diagnose intra-articular abnormalities with less postoperative pain and risk of infection. Diagnostic ankle arthroscopy is indicated for unexplained instability, pain, swelling, or hemarthrosis. Therapeutic arthroscopy is indicated for articular surface damage, cartilage and soft tissue injuries, bony impingement, osteophytes, arthrodesis, and so on.

Among various arthroscopic regions, anterior ankle arthroscopy has become increasingly popular for the diagnosis and treatment of various disorders, especially with the development of new arthroscopic instruments and techniques. There are generally three recommended portals for anterior ankle arthroscopy: the anteromedial portal (AMP), the anterocentral portal (ACP), and the anterolateral portal (ALP). The anteromedial and anterolateral portals are widely used in ankle arthroscopy [1].

Knowing the exact locations of these three portals with respect to vital structures of the ankle joint and adjacent branches and variations of neurovascular structures is critical to safely perform anterior ankle arthroscopy. However, previous studies have not described the portal locations in detail with accurate figures or statistical data.

Furthermore, previous studies have reported that the complication rate of anterior ankle arthroscopy is as high as 17%, of which more than 25% of complications involve damage to the superficial peroneal nerve [1–4]. Son et al. [5] also reported an anterior tibial artery injury rate of 4.3% during anterior ankle arthroscopy. Specifically, damage of the superficial peroneal nerve has been a focus of some authors. They believe that marking the nerve and its branches on the skin before anterior ankle arthroscopy is an important and effective way to prevent iatrogenic nerve injury [6–9]. They have described the clinical significance of the medial and intermediate dorsal cutaneous nerve during ankle arthroscopy. However, it is important to know the location of the nerve under the skin, exactly where to put the portal, even if the surgeon can identify them on the skin.

Multiple factors can affect the results of anterior ankle arthroscopy. For example, the position of nerves around the ankle joint can vary depending upon the patient's posture [2]. The nature of the procedure and anatomical variations of the vessels are also intrinsic risk factors of anterior ankle arthroscopy [10]. These anatomical variations can lead to intraoperative complications and unexpected results [5].

It is therefore necessary to investigate these anatomical variations and how the locations of neurovascular structures can change depending upon the ankle position on surgery. The aim of this study was to investigate entry points for anterior ankle arthroscopy that would minimize the risk of neurovascular injury.

## 2. Materials and Methods

**Figure 1:**
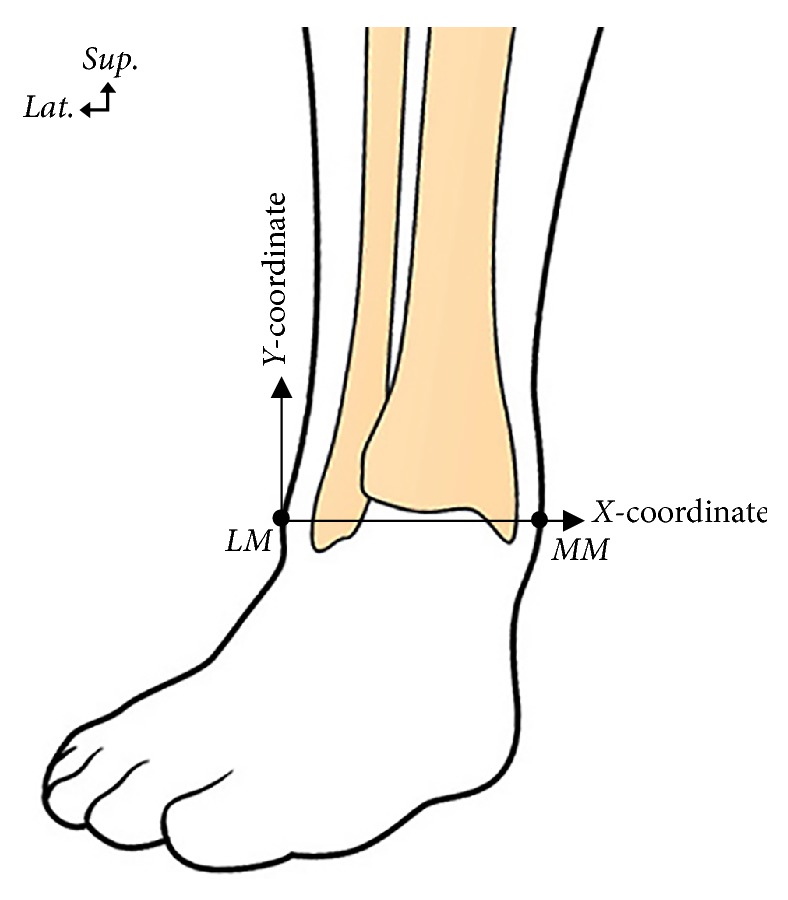
Illustration showing the reference line from the lateral malleolus (LM) to the medial malleolus (MM) and the coordinates used for localizing neurovascular and tendinous structures with respect to reference line.

Thirty-eight specimens from 21 Korean cadavers (age range from 43 to 92 years, mean age 62.3 ± 13.4 years) were used for the study; we used nonembalmed cadavers so that the position of the ankle could be changed. Cases with pathological changes or trauma to the foot and ankle were excluded. For the measurements, the most prominent point of the lateral malleolus (LM) and the medial malleolus (MM) were identified before dissection. A line connecting the LM and MM, known as the intermalleolar line, was used as a reference line. The* x*-coordinate was expressed in absolute distance along the reference line using the LM as the starting point. The* y*-coordinate was expressed in absolute distance perpendicular to the reference line. The locations of neurovascular and tendinous structures are presented with respect to the reference line (Figure 1).

All dissections were performed in the supine position. After removing the skin around the anterior ankle joint, the dissection was carefully performed to identify the nerves and veins of the superficial fascia around the ankle joint. Next, after measuring the locations of the superficial structures, they were cut and raised to identify the deep structures of the anterior ankle joint. Cadavers were maintained in the supine position during both dissection and measurement. A single observer obtained all measurements using a measuring tape and digital calipers (resolution 0.01 mm, CD-20PSX, Mitutoyo, Japan). Data were analyzed using SPSS software version 23.0 (IBM SPSS Inc., Chicago, IL, USA). The intraclass correlation coefficient was used to examine the intraobserver reproducibility of the measurements (at a confidence level of 95%).

**Figure 2:**
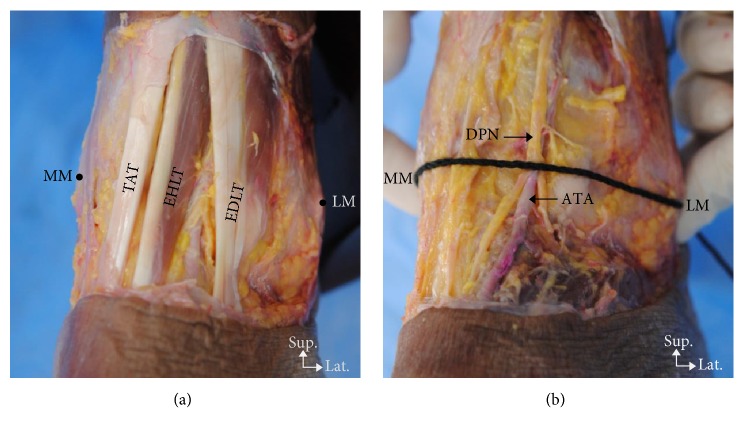
Photographs of the dissected left anterior ankle joint showing the location of the superficial and deep structures near the reference line. (a) Superficial structures: TAT, Tibialis anterior tendon; EHLT, extensor hallucis longus tendon; EDLT, extensor digitorum longus tendon; MM, medial malleolus; LM, lateral malleolus. (b) Deep structures: DPN, deep peroneal nerve; ATA, anterior tibial artery.

**Figure 3:**
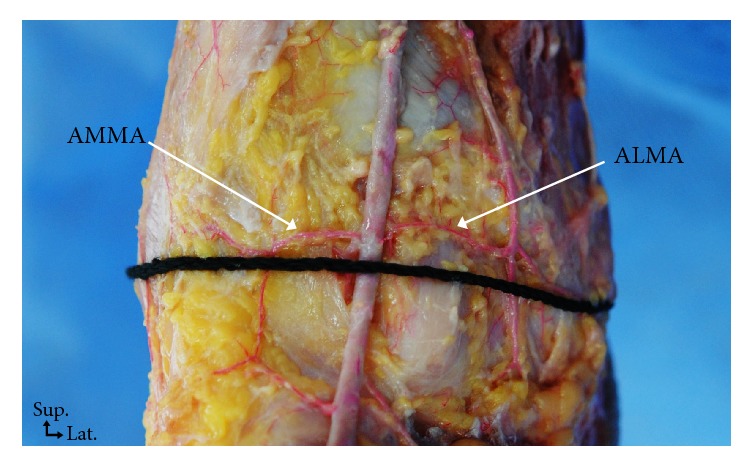
Photograph showing the branches of the anterior tibial artery. ALMA, anterior lateral malleolar artery; AMMA, anterior medial malleolar artery.

**Figure 4:**
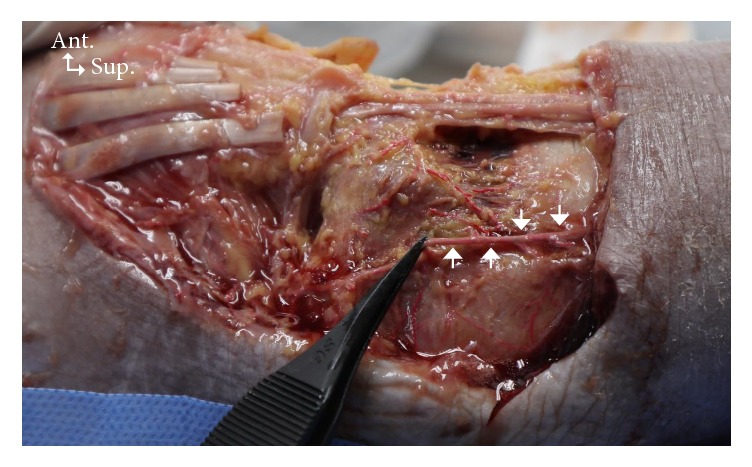
Photograph showing the perforating branch of the peroneal artery. Arrows, perforating branch of the peroneal artery; ANT, anterior; SUP, superior.

The superficial and deep structures of the anterior ankle joint were identified and measured (Figure 2). The locations of the superficial structures are as follows: (1) vein near the lateral malleolus, (2) venin near the medial malleolus, (3) medial dorsal cutaneous branch, (4) intermediate dorsal cutaneous branch, (5) saphenous nerve, (6) greater saphenous vein, (7) the 3 tendons (extensor digitorum longus, extensor hallucis longus, and tibialis anterior), and (8) the reference line (from the lateral malleolus tip to the medial malleolus tip). The locations of the deep structures are as follows: (1) perforating branch of the peroneal artery, (2) deep peroneal nerve, (3) anterior tibial artery, (4) anterior lateral malleolar artery, and (5) anterior medial malleolar artery (Figures 3 and 4).

## 3. Results

The mean distance of the reference line, from the LM to the MM, was 10.5 ± 0.8 cm. No significant difference was found in the reference line distance between males and females or between right and left ankles (*p* ≥ 0.05). The average distances on the* x*-coordinate as a percentage of the total reference line for superficial and deep structures are shown in Tables 1–5.

The greater saphenous vein and the other vein near the medial malleolus were located at 80.3% and 79.0% of the reference line from the lateral malleolus, respectively. The vein on the side medial of the GSV was located at 16.9% of the reference line from the lateral malleolus (Table 1).

The medial and intermediate dorsal cutaneous branches were located at 39.7% and 44.5% of the IML distance from the lateral malleolus, respectively. The saphenous nerve and deep peroneal nerve were located at 81.6% and 49.8% of the reference line from the lateral malleolus, respectively (Table 2).

The tendons of the extensor digitorum longus, extensor hallucis longus, and tibialis anterior muscles were located at 39.7%, 55.2%, and 65.2% of the reference line from the lateral malleolus, respectively (Table 3).

The perforating branch of the peroneal and the anterior tibial arteries crossed the reference line at 22.0% and 49.6%, respectively (Table 4). The anteromedial malleolar artery and the anterolateral malleolar artery crossed the reference line in 9 specimens (23.7%) and 8 specimens (21.0%), respectively. The location where the anterolateral malleolar artery branched out from the anterior tibial artery was on average at 2.2% above the reference line, and it crossed the reference line at 36.1% from the lateral malleolus. The anteromedial malleolar artery was on average 4.7% above the reference line, and it crossed the reference line at 57.1% from the lateral malleolus (Table 5).

## 4. Discussion

One report recently emphasized the importance of locating the superficial peroneal nerve by showing that it was damaged in more than 25% of the anterior ankle arthroscopy cases [4]. de Leeuw et al. [4] also found that using palpation with plantar flexion of the ankle to identify the superficial peroneal nerve before surgery significantly reduced the incidence of iatrogenic damage from anterior portal incisions. However, this palpation technique is not suitable for cases in which it is impossible to move the ankle or in patients with a high BMI. In this study, the medial dorsal cutaneous nerve was located at 39.7%, and the intermediate dorsal cutaneous nerve was located at 47.3% on the reference line. Also, the present study used nonembalmed cadaver specimens in the anatomic posture to closely simulate surgical conditions; therefore, these detailed anatomical results can be applied to actual patients undergoing ankle arthroscopy.

Anteromedial and anterolateral portals are widely used in ankle arthroscopy. However, little information is available regarding vascular damage during ankle arthroscopy, especially anatomical variations of the anterior tibial artery (ATA). According to a previous study [5], the ATA might be at risk from anterolateral portals during ankle arthroscopy. The present study found that the ATA and the perforating branch of the peroneal artery crossed the reference line at 49.6% and 22.0% of the reference line from the most prominent point of lateral malleolus, respectively (Table 4); the intersection points according to the* x-* and* y*-coordinates are shown in Table 5. The main trunk of the ATA crosses the middle of the reference line in most cases; however, surgeons must be mindful of the perforating branch of the peroneal artery when an anterolateral portal is attempted.

Oliva et al. reported that the intermediate dorsal cutaneous nerve was the structure most likely to be injured by the anterolateral portal [11]. Another study reported that SPN, namely, the intermediate dorsal cutaneous nerve and the medial dorsal cutaneous nerve, is one of the most common complications, accounting for up to 50% of the cases [6]. The saphenous nerve and the great saphenous vein are the most likely structures to be injured by the anteromedial portal. Our results found that the intermediate dorsal cutaneous nerve, saphenous nerve, and great saphenous vein were located at 44.5%, 81.6%, and 80.3% of the reference line from the lateral malleolus, respectively. These results could represent a valuable guide in the clinical setting.

Many patients undergo preoperative MR imaging to reduce the risk of neurovascular injury from an anterolateral portal, and a locational variant of a structure near the anterolateral portal is found in as many as 6.2% of cases [5]. Furthermore, the skill of the arthroscopic surgeon will significantly impact the surgical outcome [12]. Therefore, the positions of neurovascular structures found in this study with respect to the reference line will be helpful to surgeons regardless of their proficiency.

Some surgeons have insisted that all patients undergo topographic identification by preoperative ultrasound in order to decrease the risk of iatrogenic injury to the vessels, nerves, and tendons during ankle arthroscopy. A recent study reported a full-thickness injury to the extensor hallucis longus tendon by the anterior portal when ultrasonography was not used preoperatively [13]. Their study, however, found no cases of iatrogenic injury when preoperative ultrasonography was used. In our study, we found that the locations of the extensor digitorum longus, extensor hallucis longus, and tibialis anterior tendons were at 39.7%, 55.2%, and 65.2% of the reference line from the lateral malleolus, respectively (Table 3). These tendon locations with respect to the reference line could be helpful in cases where ultrasound equipment is unavailable.

The present study also presented the locations of veins intersecting the reference line. However, of the measured variables of the vein near the lateral malleolus and the vein near the medial malleolus, only the largest vein was measured when multiple veins were entangled with each other. The vein near the lateral malleolus, the vein near the medial malleolus, and the greater saphenous vein were located at 16.9%, 79.0%, and 80.3% of the reference line from the lateral malleolus, respectively (Table 1). Notably, the greater saphenous vein and venous plexus of medial malleolus were located very close to each other.

**Figure 5:**
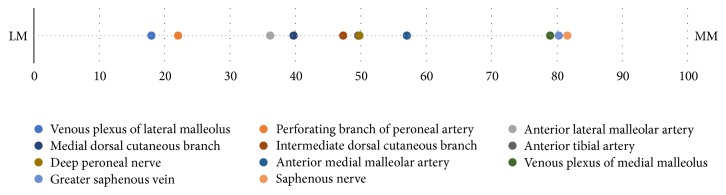
The locations of neurovascular structures on the *x*-coordinate as a percentage of the reference line. LM, lateral malleolus; MM, medial malleolus.

We found that the nerves were located at 40.0%, 50.0%, and 82.0% of the reference line from the lateral malleolus. We also found that the arteries were located at 22.0%, 35.0%, and 60% of the reference line from the lateral malleolus. If all the variables are combined (nerves, arteries, and veins), then there is no safety zone for anterior portal placement. Therefore, we recommend that surgeons concentrate primarily on the arteries and nerves in the clinical setting (Figure 5).

## Figures and Tables

**Table 1:** The mean value of the variables related vein of the superficial structures from the lateral malleolus tip and the percentage of the locations on the reference line.

Structures	Distance (*x*-coordinate)	
	Mean ± SD (cm)	Mean ± SD (%)
Venous plexus of lateral malleolus	1.9 ± 1.3	18.0 ± 11.8
Venous plexus of medial malleolus	8.2 ± 1.2	79.0 ± 8.4
Greater saphenous vein	8.5 ± 0.7	80.3 ± 5.9

**Table 2:** The mean value of the variables related nerve of the superficial structures from the lateral malleolus tip and the percentage of the locations on the reference line.

Structures	Distance (*x*-coordinate)	
	Mean ± SD (cm)	Mean ± SD (%)
Medial dorsal cutaneous branch	4.2 ± 1.1	39.7 ± 10.5
Intermediate dorsal cutaneous branch	5.0 ± 0.9	47.3 ± 9.3
Saphenous nerve	8.3 ± 0.8	81.6 ± 7.1

**Table 3:** The mean value of the variables related tendon of the superficial structures from the lateral malleolus tip and the percentage of the locations on the reference line.

Structures	Distance (*x*-coordinate)	
	Mean ± SD (cm)	Mean ± SD (%)
Extensor digitorum longus tendon	4.2 ± 0.5	39.7 ± 3.2
Extensor hallucis longus tendon	5.8 ± 0.5	55.2 ± 3.8
Tibialis anterior tendon	6.9 ± 0.6	65.2 ± 4.4

**Table 4:** The mean value of the variables on deep structures from the lateral malleolus tip and the percentage of the locations on the reference line.

Structures	Distance (*x*-coordinate)	
	Mean ± SD (cm)	Mean ± SD (%)
Perforating branch of peroneal artery	2.3 ± 0.7	22.0 ± 6.5
Deep peroneal nerve	5.2 ± 0.5	49.8 ± 4.5
Anterior tibial artery	5.2 ± 0.6	49.6 ± 4.7

**Table 5:** The mean value of the deep structures according the *x* and *y* coordinates and the percentage of the locations on the reference line.

Structures	Intersectional point on the reference line (*x*-coordinate)		Branching out point from the anterior tibial artery (*y*-coordinate)
	Mean ± SD (cm)	Mean ± SD (%)	Mean ± SD (cm)
Anterolateral malleolar artery	3.7 ± 0.8	36.1 ± 6.57	0.2 ± 0.4
Anteromedial malleolar artery	6.0 ± 0.6	57.1 ± 5.7	0.5 ± 0.3

## References

[1] Golanó P., Vega J., de Leeuw P. A. J. (2016). Anatomy of the ankle ligaments: a pictorial essay.

[2] Chamseddin K. H., Kirkwood M. L. (2016). Anterior tibial artery pseudoaneurysm following ankle arthroscopy in a hemophiliac patient.

[3] Deng D. F., Hamilton G. A., Lee M., Rush S., Ford L. A., Patel S. (2012). Complications Associated with Foot and Ankle Arthroscopy.

[4] de Leeuw P. A. J., Golanó P., Blankevoort L., Sierevelt I. N., van Dijk C. N. (2016). Identification of the superficial peroneal nerve: Anatomical study with surgical implications.

[5] Son K.-H., Cho J. H., Lee J. W., Kwack K.-S., Han S. H. (2011). Is the anterior tibial artery safe during ankle arthroscopy?: Anatomic analysis of the anterior tibial artery at the ankle joint by magnetic resonance imaging.

[6] Suzangar M., Rosenfeld P. (2012). Ankle Arthroscopy: Is Preoperative Marking of the Superficial Peroneal Nerve Important?.

[7] Ferkel R. D., Small H. N., Gittins J. E. (2001). Complications in foot and ankle arthroscopy.

[8] de Leeuw P. A. J., Golanó P., Sierevelt I. N., van Dijk C. N. (2010). The course of the superficial peroneal nerve in relation to the ankle position: Anatomical study with ankle arthroscopic implications.

[9] Rodeo S. A., Forster R. A., Weiland A. J. (1993). Current concepts review: Neurological complications due to arthroscopy.

[10] Bonnin M., Bouysset M. (1999). Arthroscopy of the ankle: Analysis of results and indications on a series of 75 cases.

[11] Oliva X. M., Méndez López J. M., Monzo Planella M., Bravo A., Rodrigues-Pinto R. (2015). Anatomical relations of anterior and posterior ankle arthroscopy portals: a cadaveric study.

[12] Barg A., Saltzman C. L., Beals T. C., Bachus K. N., Blankenhorn B. D., Nickisch F. (2016). Arthroscopic Talar Dome Access Using a Standard Versus Wire-Based Traction Method for Ankle Joint Distraction.

[13] Scheibling B., Koch G., Clavert P. (2017). Cadaver study of anatomic landmark identification for placing ankle arthroscopy portals.

